# Adaptive Introgression: An Untapped Evolutionary Mechanism for Crop Adaptation

**DOI:** 10.3389/fpls.2019.00004

**Published:** 2019-02-01

**Authors:** Concetta Burgarella, Adeline Barnaud, Ndjido Ardo Kane, Frédérique Jankowski, Nora Scarcelli, Claire Billot, Yves Vigouroux, Cécile Berthouly-Salazar

**Affiliations:** ^1^Institut de Recherche pour le Développement, UMR DIADE, Montpellier, France; ^2^DIADE, Université de Montpellier, Montpellier, France; ^3^Centre de Coopération Internationale en Recherche Agronomique pour le Développement, UMR AGAP, Montpellier, France; ^4^AGAP, Université de Montpellier, Centre de Coopération Internationale en Recherche Agronomique pour le Développement, Institut National de la Recherche Agronomique, Montpellier SupAgro, Montpellier, France; ^5^Laboratoire National de Recherches sur les Productions Végétales, Institut Sénégalais de Recherches Agricoles, Dakar, Senegal; ^6^Laboratoire Mixte International Adaptation des Plantes et Microorganismes Associés aux Stress Environnementaux, Dakar, Senegal; ^7^Centre de Coopération Internationale en Recherche Agronomique pour le Développement, UPR GREEN, Montpellier, France; ^8^GREEN, Centre de Coopération Internationale en Recherche Agronomique pour le Développement, Université de Montpellier, Montpellier, France; ^9^Bureau d’Analyses Macro-Economiques, Institut Sénégalais de Recherches Agricoles, Dakar, Senegal

**Keywords:** crops, wild relatives, domestication, selection, gene flow, adaptive introgression, farmer’s practices

## Abstract

Global environmental changes strongly impact wild and domesticated species biology and their associated ecosystem services. For crops, global warming has led to significant changes in terms of phenology and/or yield. To respond to the agricultural challenges of this century, there is a strong need for harnessing the genetic variability of crops and adapting them to new conditions. Gene flow, from either the same species or a different species, may be an immediate primary source to widen genetic diversity and adaptions to various environments. When the incorporation of a foreign variant leads to an increase of the fitness of the recipient pool, it is referred to as “adaptive introgression”. Crop species are excellent case studies of this phenomenon since their genetic variability has been considerably reduced over space and time but most of them continue exchanging genetic material with their wild relatives. In this paper, we review studies of adaptive introgression, presenting methodological approaches and challenges to detecting it. We pay particular attention to the potential of this evolutionary mechanism for the adaptation of crops. Furthermore, we discuss the importance of farmers’ knowledge and practices in shaping wild-to-crop gene flow. Finally, we argue that screening the wild introgression already existing in the cultivated gene pool may be an effective strategy for uncovering wild diversity relevant for crop adaptation to current environmental changes and for informing new breeding directions.

## Introduction

The fate of wild and domesticated species and their associated ecosystem services is increasingly depending on global environmental changes, as climate warming, nitrogen cycle alteration or land use (Walther et al., 2002; Perring et al., 2015; Shibata et al., 2015). For agriculture, great concerns are caused by the potential decrease in productivity and increase of losses at post-harvesting stages (Beddington, 2010). For example a decline by 4–6% of global maize and wheat yield has been registered since the early 1980s (Lobell et al., 2011), and up to 30% in response to extreme year conditions (Ciais et al., 2005). Future climate scenarios foresee an acceleration of the rise in temperature and an increase in rainfall variability (IPCC, 2014), which are probably the prelude to further dramatic consequences for agricultural supply worldwide (Wheeler and von Braun, 2013). The issue is particularly worrying for fast population growing developing countries of Africa and Asia, where average yield of major crops as wheat, maize, sorghum, and pearl millet may decline up to 40% in the worst predicted scenarios (Schlenker and Lobell, 2010; Knox et al., 2012; Sultan et al., 2013). Noteworthy, yield loss might be exacerbated by higher insects damages (Deutsch et al., 2018).

Different strategies for adapting worldwide agriculture can be considered. In Africa, 75% of countries will have novel climates with analogs in the current climate of other countries, suggesting that international movement of germplasm could be part of the mitigation strategy (Burke et al., 2009). However, future climate models also predict a reduction up to nearly 60% in areas suitable for agriculture (Rippke et al., 2016), meaning that they will be a need for crops able to grow in the novel “unsuitable” ones. Studies comparing the efficacy of different agricultural adaptations for West Africa agriculture showed that increasing stress resistance of varieties would be a more beneficial strategy than changes in agricultural practices (Guan et al., 2017; Parkes et al., 2018). Increasing crops ability to cope with biotic and abiotic stresses will likely play a major role for adapting agriculture to climate changes in the next decades.

However, the genetic diversity of crop species may provide a too narrow base for the evolution of new adaptations at the speed and magnitude required by current changes. Domesticated species usually harbor a reduced genetic diversity compared to their wild counterparts, a consequence of the recurrent rounds of selection applied to the ancestral wild species during domestication and successive breeding/improvement processes (Glémin and Bataillon, 2009; Meyer and Purugganan, 2013). Wheat, the most widely cultivated crop on earth (FAO, 2016), has lost more than 70% of diversity compared to its wild progenitor, the wild emmer (Haudry et al., 2007). On the other hand, the later carries significant diversity for biotic and abiotic resistances (Huang et al., 2016). The interest of the wild reservoir as source of readily available adaptations to climate change for crops is now largely acknowledged (Warschefsky et al., 2014; Dempewolf et al., 2017). As a result, a considerable number of studies look at adaptive genes in crop wild relatives (Berthouly-Salazar et al., 2016; Fustier et al., 2017; Brunazzi et al., 2018; von Wettberg et al., 2018) with the aim of identifying genes that could be introduced in crops by artificial breeding schemes (Hajjar and Hodgkin, 2007). However, another mechanism allowing to rapidly acquire new variants that is largely overlooked in crop research for breeding is spontaneous gene flow.

Gene flow is the exchange of genetic material between different genetic pools either from the same or different species (Slatkin, 1985). In this definition, gene flow includes hybridization, the mating between individuals from populations ‘distinguishable on the basis of one or more heritable characters’ (Arnold, 2004). Gene flow can result in an increase of additive variance up to two or three orders of magnitude more than that introduced by mutation in the same time lapse (Grant and Grant, 1994) and, thus, may amount to an immediate primary source of functional alleles (Ellstrand, 2014). A long-term outcome of gene flow is introgression, that occurs when a foreign variant is permanently incorporated in the local gene pool through back-crossing (Anderson and Hubricht, 1938). Foreign functional variants that increases the fitness of the recipient pool are often referred to as “adaptive introgression”. Compared to neutral introgression, which could be lost by drift across generations, adaptive introgression is maintained by selection and may eventually give rise to fixation. An important feature of gene flow by hybridization is the potential to introduce large sets of new alleles simultaneously at multiple unlinked loci, which allows adaptation even for polygenic traits (Mallet, 2007; Abbott et al., 2013) and can thus promote rapid species evolution (e.g., Arnold and Martin, 2009; Hedrick, 2013; Arnold and Kunte, 2017). The role of introgression for species adaptation and evolution has been early recognized by evolutionary biologist (Anderson, 1953; Rieseberg and Wendel, 1993) and has gained momentum in the last years thanks to the possibility to explore it at the genomic level.

In the context of species conservation and management a large body of literature discuss how gene flow could also be associated with negative effects. Among undesirable consequences of gene flow from a species conservation and management point of view, there are the evolution of invasiveness (Ellstrand and Schierenbeck, 2000; Whitney et al., 2006), transgene escape (Ellstrand et al., 2013 and references therein), or the genetic erosion of native populations (Wolf et al., 2001) potentially leading to extinction (Todesco et al., 2016). Comparatively less attention has been paid to the potential benefits of managed gene flow to increase genetic variation for species rescue (Hedrick, 2009) and adaptation (Aitken and Whitlock, 2013). Up to now, the potential of adaptive introgression as a source of adaptation to on-going global changes has been overlooked (Suarez-Gonzalez et al., 2018b), notably for domesticated species.

In this paper, we focus on adaptive introgression in domesticated plants and the interest of this mechanism for crop adaptation to environmental changes. For this, we reviewed studies of adaptive introgression to examine how often this process has been documented in domesticated plant species and which are the most commonly used approaches to study it. We focused on sexually reproductive organisms and we did not directly discuss horizontal gene transfer (for a review on this topic see Arnold and Kunte, 2017). We also excluded cases of artificial crossing in the context of modern breeding. Within this framework, we screened several case studies addressing adaptive introgression and we selected 39 published works representing the most convincing cases in our opinion (Table 1). Works were retained when they showed evidence of both introgression and selection on the introgression, on the basis of statistical analyses or knowledge of the implication of the target trait in adaptation; note that the two lines of evidence could have been gathered in different studies. We first discuss some examples of adaptive introgression included in Table 1, with a focus on domesticated plants. Next, we discuss the methodological approaches and challenges to detecting adaptive introgression, notably with genetic data. We finally focus on the agronomic and genetic circumstances influencing the frequency of adaptive introgression into crop genetic pools and discuss the opportunities provided by this spontaneous phenomenon for the improvement of crops in the context of current environmental changes.

**Table 1:** Summary of studies reviewed.

Group	Donor	Recipient	Data	Method for detection of introgression	Method for detection of Selection	Adaptive trait	Publication
Animal	*Anopheles gambiae*	*A. coluzzi*	Genomic data	Diagnostic alleles	Haplotype based test; test for temporal evolution of allele frequencies	Pesticide resistance	Lynd et al., 2010
Animal	*Anopheles gambiae*	*A. coluzzi*	Genomic data	Diagnostic alleles	Differentiation approach; diversity statistics	Pesticide resistance	Norris et al., 2015
Animal	*Drosophila yakuba*	*D. santomea*	Genomic data (mtDNA)	Genes genealogy; isolation with migration model	Coalescent simulations	*na*	Llopart et al., 2014
Animal	*Mus spretus*	*M. m. domesticus*	Genomic and phenotypic data	Genes genealogy; Hudson–Kreitman–Aguade test	Differentiation approach; fitness measures	Pesticide resistance	Song et al., 2011
Animal	*Mus spretus*	*M. m. domesticus*	Genomic data	Genes genealogy	XP-CLR	Pesticide resistance	Liu et al., 2015
Animal	*Mus musculus musculus*	*M. m. domesticus*	Genomic data	Local ancestry inference	XP-CLR; differentiation approach; coalescent simulations	Genetic disease, alpha-amylase genes	Staubach et al., 2012
Animal	*Oncorhynchus mykiss*	*O. clarkii lewisi*	Genomic data	Diagnostic alleles	Heterogeneity test of Long (1991)	Fecundity	Hohenlohe et al., 2013
Animal	*Ambystoma tigrinum mavortium*	*A. californiense*	Genomic data	Diagnostic alleles	Heterogeneity test of Long (1991)	*na*	Fitzpatrick et al., 2009
Animal	*Sus* sp.	*Sus scrofa domesticus*	Genomic data	Genes genealogy	Differentiation approach	Highland adaptation	Ai et al., 2015
Animal	*Sus celebensis*	*Sus scrofa domesticus*	Genomic data	Genes genealogy; differentiation statistics	Differentiation approach	Aggressive behavior	Zhu et al., 2017
Animal	*Canis lupus familiaris*	*C. lupus lupus*	Genomic data	Genes genealogy	Haplotype based test	Concealment during predation	Anderson et al., 2009
Animal	*C. lupus lupus*	*C. l. familiaris*	Genomic data	D statistic	Haplotype based test; differentiation outlier approach	Highland adaptation	Miao et al., 2016
Animal	*Ovis aries*	*Ovis aries*	Genomic data	Local ancestry inference; populations genealogy	Differentiation outlier approach	*na*	Rochus et al., 2018
Animal	*Anguilla rostrata*	*A. anguilla*	Genomic data	Local ancestry inference	Differentiation outlier approach; allele frequencies outlier test	*na*	Gagnaire et al., 2009
Animal	*Heliconius melpomene*	*H. cydno clade*	Genomic data	Genes genealogy; isolation with migration model, linkage disequilibrium analysis	Not addressed, but trait previously tested as under natural selection	Wing pattern	Pardo-Diaz et al., 2012
Animal	*Heliconius melpomene*	*H. beskei*	Genomic data	Gene genealogy; D-statistic and *f-*statistics	Not addressed, but trait previously tested as under natural selection	Wing pattern	Zhang et al., 2014
Animal	*Heliconius melpomene*	*H. cydno clade*	Genomic data	Gene genealogy; D-statistic and *f-*statistics	Not addressed, but trait previously tested as under natural selection	Wing pattern	Enciso-Romero et al., 2017
Animal	*Lepus californicus*	*L. americanus*	Genomic and phenotypic data	Phylogenetic analysis; differentiation statistics; *f*-statistics; coalescent simulations	Composite likelihood ratio (CLR) test; estimation of selection coefficient	Winter-brown-color coat	Jones et al., 2018
Human	*Homo s. denisovans*	*H. s. sapiens*	Genomic data	D statistic, S^∗^ statistic	Differentiation outlier approach	Highland adaptation	Huerta-Sánchez et al., 2014
Human	*Homo s. neanderthalensis, H. s. denisovans*	*H. s. sapiens*	Genomic and expression data	Diagnostic alleles	McDonald–Kreitman test; haplotype based test; differentiation outlier approach	Immune response	Deschamps et al., 2016
Human	*Homo neanderthalensis, H. s. denisovans*	*H. s. sapiens*	Genomic and expression data	Differentiation comparisons; haplotype length vs. ILS (incomplete lineage sorting)	Differentiation outlier approach; gene expression; genotype–phenotype association	Immune response	Dannemann et al., 2016
Human	*Homo neanderthalensis, H. s. denisovans*	*H. s. sapiens*	Genomic data	*F* statistics, S^∗^ statistic	Coalescent simulations	Immune response and metabolism	Vernot et al., 2016
Human	*Homo neanderthalensis, H. s. denisovans*	*H. s. sapiens*	Genomic data	Diagnostic alleles	Coalescent simulations	Immune response, defense, regulatory regions, pigmentation	Gittelman et al., 2016
Human	*Homo neanderthalensis, H. s. denisovans*	*H. s. sapiens*	Genomic data	*f* statistics, diagnostic alleles, local ancestry inference	Differentiation outlier approach	Cold tolerance	Racimo et al., 2017
Human	*Homo s. neanderthalensis*	*H. s. sapiens*	Genomic data	Genes genealogy	Allele frequencies outlier test	Immune response	Mendez et al., 2012
Human	*Homo s. neanderthalensis*	*H. s. sapiens*	Genomic data	Diagnostic alleles	Coalescent simulations; haplotype based test	Immune response	Sams et al., 2016
Human	*Homo s. neanderthalensis*	*H. s. sapiens*	Genomic data	Diagnostic alleles	Differentiation outlier approach; haplotype based test; XP-CLR; coalescent simulations	Immune response	Quach et al., 2016
Human	*H. s. sapiens*	*H. s. sapiens*	Genomic data	Population genealogy; D statistic and *f* statistics	Allele frequencies outlier test	Highland adaptation	Jeong et al., 2014
Plant	*Arabidopsis lyrata*	*A. arenosa*	Genomic data	*f* statistics	Differentiation outlier approach	Serpentine syndrome	Arnold et al., 2016
Plant	*Helianthus debilis*	*H. annuus*	Phenotypic data	Experimental hybrid populations	Common garden experiments – Fitness measures	Herbivory, drought	Whitney et al., 2006, 2010
Plant	*Helianthus debilis*	*H. annuus*	Genomic and phenotypic data	Experimental hybrid populations	Genotype–phenotype association – Fitness measures	Number of seeds and pollen export	Whitney et al., 2015
Plant	*Iris fulva*	*I. brevicaulis*	Genomic and phenotypic data	Experimental hybrid populations	Genotype–phenotype association – Fitness measures	Flood tolerance	Martin et al., 2006
Plant	*Populus balsamifera*	*P. trichocarpa*	Genomic, expression and phenotypic data	Local ancestry inference	Diversity statistics; genotype–phenotype association	Light response	Suarez-Gonzalez et al., 2016
Plant	*Populus balsamifera*	*P. trichocarpa*	Genomic, expression and phenotypic data	Local ancestry inference	Diversity statistics	Disease resistance	Suarez-Gonzalez et al., 2018a
Plant	*Zea mays mexicana*	*Z. m. mays*	Genomic data	Local ancestry inference	Genotype–environment association	Highland adaptation	Hufford et al., 2013
Plant	*Oryza sativa japonica*	*Oryza sativa indica*	Genomic data	Diagnostic alleles	Haplotype based test	Fragrance	Kovach et al., 2009
Plant	*Senecio squalidus*	*S. vulgaris*	Genomic data	Diagnostic alleles	Not addressed but high related fitness trait	Flower asymmetry	Kim et al., 2008
Plant	*Arabidopsis halleri*	*A. lyrata*	Genomic data	Differentiation comparisons; isolation with migration model	Not addressed but high related fitness trait	Pistil self-incompatibility	Castric et al., 2008
Plant	*Solanum microdontum* or other wild species	*S. tuberosum*	Genomic data	Genes genealogy	Not addressed, but trait previously tested as under natural selection	Long-day-maturity phenotype	Hardigan et al., 2017

*Species names for donor and recipient taxa are listed, as well as the type of data and methods used for (1) detection of the introgression and (2) detection of the selection. “Genomic data” term include s whole-genome sequences or candidates genes sequencing. “Genetic data refers to molecular markers such as QTL or SSR.”*

## Empirical Evidence of Adaptive Introgression

Despite the occurrence of hybridization in nature, estimated to involve 25% of plants and 11% of animals in the interspecific case (Mallet, 2005), relatively little experimental evidence exists of adaptation and enhanced fitness associated with an introgressed trait, what constitutes an adaptive introgression. This may be because investigating the fitness of introgression is intrinsically difficult. For example, a well-known evidence of adaptive introgression in the annual sunflowers (*Helianthus* genus) was based on crossing and backcrossing cycles to experimentally reproduce introgression and demonstrate the increase of fitness in the recipient taxon (Whitney et al., 2006, 2010). However, in the last years, the quest for adaptive introgression has been facilitated with the access provided by high-throughput sequencing technologies to the genome of virtually any species. Researchers can now search for selection signatures on the introgressed variant in the recipient genomes with population genetics and phylogenetic approaches (e.g., Racimo et al., 2015; Arnold et al., 2016). An increasing number of publications involving large-scale genetic data are accumulating in this field (Table 1). These studies reveal or confirm instances of adaptive introgression in many kinds of organisms, including humans (e.g., Reich et al., 2009; Green et al., 2010; Huerta-Sánchez et al., 2014; Sankararaman et al., 2014; Hsieh et al., 2016), animals (e.g., insects, Weetman et al., 2010; Enciso-Romero et al., 2017; rodents, Song et al., 2011; Staubach et al., 2012). From these works, it also appears that different abiotic and biotic selective pressures drive the introgression of adaptive traits, with evolutionary consequences spanning different spatial and temporal scales.

In crop evolution studies, it is increasingly appreciated that gene flow have contributed to shape the genome diversity of most domesticated species, either plants or animals. Domestication has likely been a protracted process, in which the domesticated forms differentiated from their wild ancestors diffused geographically while continuing to exchange genetic material with local wild or cultivated relatives (Allaby et al., 2008; Meyer and Purugganan, 2013). Genetic surveys support this scenario. Evidence of historical and current gene flow have been reported in several crop species, like cereals (e.g., barley, Poets et al., 2015; maize, Matsuoka et al., 2002; rice, Choi et al., 2017; pearl millet, Burgarella et al., 2018), legumes (e.g., common bean, Rendón-Anaya et al., 2017; soybean, Han et al., 2016), and tree species (e.g., apples, Cornille et al., 2012; Ma et al., 2017; grapes, Myles et al., 2011; olives, Diez et al., 2015). In some cases, the signatures of introgression are pervasive within the genome of domesticated forms, which appears to be a mosaic of fragments originating from different cultivated and wild populations (Pankin and von Korff, 2017; Pankin et al., 2018).

While proofs of introgression have been widely gathered, few examples of adaptive genetic exchanges have documented the implication of introgression in the adaptive evolution of domesticated and wild-relative populations. In some cases, the adaptive introgression concerns domesticated traits (e.g., in rice, Choi et al., 2017; or sheeps, Rochus et al., 2018), in others, traits related to the response to abiotic and biotic stressors (e.g., in maize, Hufford et al., 2013; or dogs, Miao et al., 2016). In maize, the potential adaptive outcome of introgression is the adaptation to altitude acquired by highland landraces from wild populations. Maize was domesticated from low altitude wild populations of teosinte (*Zea mays* ssp. *parviglumis*) and colonized high altitude environments where gene flow with a different wild relative (*Z*. *m*. *mexicana*) occurs (Matsuoka et al., 2002). Hufford et al. (2013) performed genome scans on Mexican sympatric populations of maize and *mexicana* and found nine genomic regions of introgression of *mexicana* into maize landraces. These regions related to adaptive traits such as the quantity of leaf macrohairs and pigmentation intensity, could have helped maize to adapt to high altitude (Hufford et al., 2013). Likewise, potato (*Solanum tuberosum*) diversification may have been triggered by the introgression of wild alleles of the *StCDF1* gene, which disables the circadian regulation enabling tuberization under long days. Wild *StCDF1* variants are adaptive in non-tropical regions and may have allowed the cultivation of *S. tuberosum* in Europe or N-America (Hardigan et al., 2017). For pearl millet (*Cenchrus americanus*), a major staple African cereal, gene flow from wild divergent populations significantly increased the genetic diversity of the cultivated gene pool and possibly lead to introgression of local adaptations (Burgarella et al., 2018).

The examples reported above concern mainly the introgression of adaptive traits from wild to crop populations, but adaptive introgression has been suggested also between domesticated forms. An example is provided by Asian rice. Complex introgressive gene flow would have shuffled the genome of Asian rice, leading to the current main groups *O. sativa japonica* and *O. s. indica* (Choi et al., 2017, but see Civáň and Brown, 2018). According to some authors, *O. s. indica* had acquired major domestication alleles indirectly, through gene flow from the domesticated *O. s. japonica* into the wild progenitor *O. rufipogon* or into putative proto-*indica* populations (Choi et al., 2017). White pigmentation, aromatic fragrance and glutinous starch are some of the phenotypic traits involved in such allele transfers driven by the directional selection associated with local cultural preferences (Olsen and Purugganan, 2002; Kovach et al., 2009; Huang et al., 2012).

In contrast to previous examples, which regard adaptive introgression shaping the domestication phenotype, adaptive traits can move in the opposite direction, from the fields to the wild. The uncontrolled escape of agricultural adaptations (e.g., resistance to biotic and abiotic stressors, often achieved with transgenes) from fields to wild populations is a potential case of adaptive introgression that takes place at very short evolutionary time scale (e.g., Pilson and Prendeville, 2004; Ellstrand et al., 2013). Several studies have investigated to what extent introgression of crop alleles could increase the invasiveness of weedy populations in different crop-wild systems (Hooftman et al., 2007; Rose et al., 2009; Uwimana et al., 2012). An example is the transfer of imidazolinone herbicides resistance from rice cultivars into weedy populations, which took very few years and led to significant economic loss for farmers (Merotto et al., 2016).

## Characterizing Adaptive Introgression With Genetic Data

To infer adaptive introgression, it is necessary to demonstrate (1) the introgression, by showing the foreign origin of the genetic variant and its persistence in the recipient pool (i.e., should be found in backcrossed generations), and (2) its adaptive value, by identifying selection footprints on the introgressed fragment and, ideally, its fitness value. Genomic studies of adaptive introgression seek to aim at gathering these two lines of evidence. A variety of genomic patterns can be observed in the recipient population due to the multiples factors (migration rate, number of generations since introgression, intensity of selection) that affect the introgression process and its interaction with selection. As these are complex patterns, there is no unique approach to detecting signatures of adaptive introgression (Table 1).

### Detection of Introgression

The aim of detecting introgression is to identify populations and individuals of admixed origin and quantify rates of gene flow, but also to find the traits or the genomic regions that have crossed isolation barriers. The availability of whole genome data maximizes the chances of detecting introgression even when it is rare in the genome (Hufford et al., 2013; Racimo et al., 2015; Schaefer et al., 2016; Rochus et al., 2018). We describe approaches used to detect introgression with genetic data, bearing in mind that none of them provides absolute proof of introgression and that an effective strategy can be to gather evidence in different ways.

**FIGURE 1:**
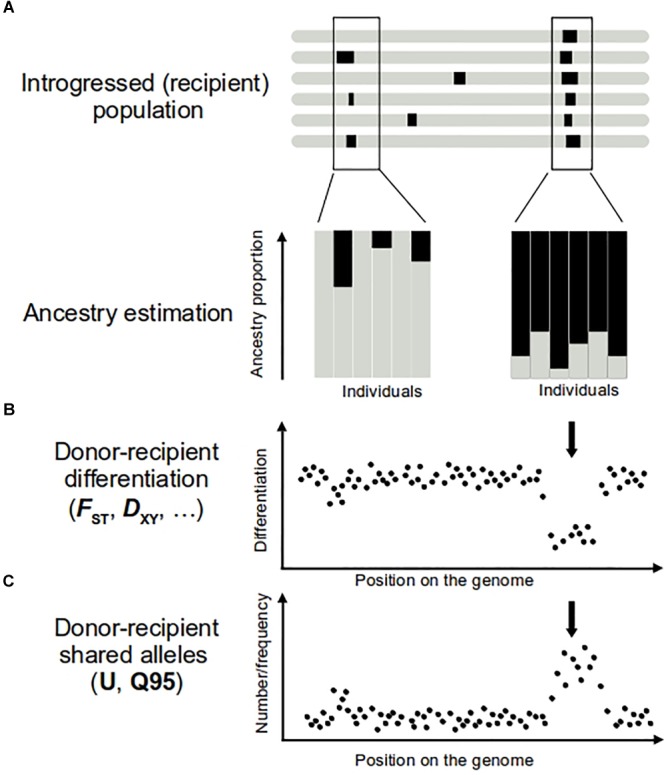
Approaches to detect introgression **(A,B)** and adaptive introgression **(C)**. On top representation of several genomes of the introgressed population, with introgression regions represented in black. Regions of donor origin in the recipient genome can be revealed by performing ancestry analyses **(A)** and comparisons of donor–recipient differentiation levels **(B)**. Individuals bearing introgression show an ancestry origin in the donor population (**A**, in black). In the case of adaptive introgression, a large proportion of individuals of the recipient population show ancestry from donor population (**A**, right), while only few of them show a donor ancestry in the genome region carrying a neutral introgression (**A**, left). In the adaptive introgression, donor–recipient differentiation is lower (**B**, arrow) than the mean genome value. Positive selection increases the frequency of the donor allele and the neutral variants physically linked to it. The result is a higher number and frequency of alleles shared by donor and recipient populations (**C**, arrow) in this part of the genome. To calculate U and Q_95_ statistics, another condition should be met, that pattern described in **(C)** are absent in other non-introgressed populations.

The ability to detect introgression increases with the divergence between the hybridizing taxa. For higher divergent taxa, there are more markers fixed between species or with large allele frequency differences. These “diagnostic alleles” allow easy identification of the ancestry of a genomic fragment in the recipient population (e.g., Smith et al., 2004; Kim et al., 2008; Kovach et al., 2009; Norris et al., 2015; Gittelman et al., 2016). However, even with slight differences in allele frequencies, ancestry estimations can be performed to identify genetically intermediate individuals potentially indicative of introgression. A variety of approaches are available for this task. “Global ancestry” inferences provide estimations of different population contributions averaged across the genome (Padhukasahasram, 2014) (Figure 1A). Their power to detect gene flow comes from the use of multiple independent (i.e., not physically linked) polymorphic markers. Such approaches include multivariate analyses (e.g., Patterson et al., 2006; Jombart et al., 2009) or model-based clustering algorithms (e.g., Pritchard et al., 2000; Anderson and Thompson, 2002; Alexander et al., 2009). These methods have been applied both genome-wide (Gagnaire et al., 2009; e.g., Rochus et al., 2018) and to single genomic regions. For example, window-based analyses of global ancestry along the maize genome had proven useful to identify introgressed fragments from the wild progenitor teosinte (Hufford et al., 2013).

When higher density molecular markers are available, “local ancestry” inferences (Padhukasahasram, 2014) are able to assign an ancestry probability to each polymorphic variant (Racimo et al., 2015; Schaefer et al., 2016). Local ancestry methods use probabilistic approaches, such as Hidden Markov Models (e.g., Reich et al., 2012), or Conditional Random Fields (e.g., Sankararaman et al., 2014) to infer the ancestry state of each site, taking into account the information of physically close positions. As physical linkage disequilibrium patterns dilute with generations, these approaches are less efficient for the detection of ancient introgression, compared to global ancestry methods. While some implementations require phased data (e.g., Song and Hein, 2005) or training data (e.g., Sankararaman et al., 2014), more recent developments have overcome these constraints (e.g., Guan, 2014). So far, such approaches have been mainly applied to model species (e.g., Staubach et al., 2012 on *Mus musculus*; Turissini and Matute, 2017 on *Drosophila*; Zhou et al., 2016 on humans), but the increasing availability of whole genome data will soon make them suitable for other study systems.

**FIGURE 2:**
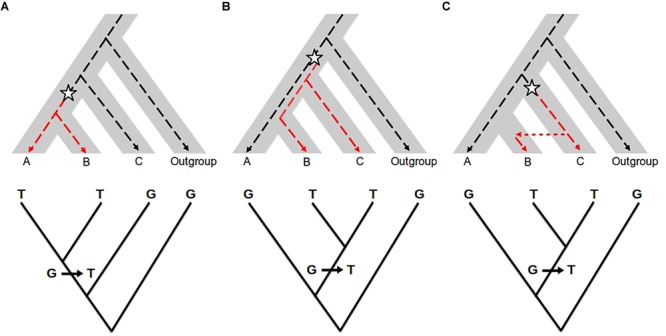
Effect of introgression and incomplete lineage sorting (ILS) in molecular phylogenetics. Top: The species (or population) tree is represented by the gray area. The dotted line represents a single gene genealogy. The star represents a mutation changing the ancestral allele G (black dotted line) into the derived allele T (red dotted line). Bottom: Gene genealogy inferred from molecular data. **(A)** Congruent gene genealogy with species/population tree; **(B)** ILS: ancestral polymorphism is maintained before the divergence between A and B, so that B shares the allele T with C and not with A; **(C)** Introgression: B receives the allele T from C by gene flow. In the case of ILS and introgression, the gene genealogy (bottom) is not consistent with the species/population tree but similar between the two. The two processes cannot be distinguished from each other when only using gene genealogy approaches.

The approaches described above help to quantify the amount of shared diversity between genetic pools. Shared variants between populations may be the result of different processes other than introgression: the retention of ancestral polymorphic alleles by chance (referred to as Incomplete Lineage Sorting, ILS, Figure 2), balancing selection or convergence (see Hedrick, 2013 for a comparison). For lower divergence times (such as for many wild-crop complexes), the probability that the two related groups have conserved ancestral polymorphism is higher. Thus, in most cases, the main challenge to detecting introgression is to distinguish it from ancestral shared polymorphism. Tracking the absence of the introgressed variants in ancient samples of the recipient pool would be an efficient way of excluding shared ancestral polymorphism. However, historical samples are difficult to obtain for most biological systems, so different methods have been developed to search for specific signatures on the genome that help to differentiate between introgressed fragments and inherited ancestral fragments.

Coalescent samplers have been widely used to test for gene flow versus ILS using maximum likelihood or Bayesian models (Pinho and Hey, 2010). However, they are not straightforwardly applied to all study systems, because they require a strong computation effort and are not easy to transpose to a genome-wide scale. An alternative simpler strategy takes advantage of the expectations associated with the phylogenetic relationships between individuals or populations (Figure 2). Given a genealogical tree describing the history of divergence between taxa, a precise amount of shared variation between branches is expected because of drift and ILS. A significant excess of shared variation instead may be indicative of gene flow (Kulathinal et al., 2009; Patterson et al., 2012; Peter, 2016). A number of statistics have been developed to test for the excess of shared polymorphism. The most used are the D-statistic (or ABBA-BABA test, Green et al., 2010; Durand et al., 2011) and the *f3* and *f4* statistics (globally referred to as *f*-statistics, Reich et al., 2009, 2012). These statistics were initially developed to analyze human populations and have proven to be useful in other study systems, e.g., to detect the introgression of adaptation to serpentine soils in *Arabidopsis arenosa* (Arnold et al., 2016). In general, the power of these tests to detect admixed genomes or populations is greater when applied to genome-wide data (see Patterson et al., 2012 for a review; Peter, 2016), but most recent statistics can be applied to small genomic regions, e.g., *f*_D_ (Martin et al., 2015; Racimo et al., 2017).

Other approaches take advantage of haplotype characteristics to distinguish between introgression and ILS. As recombination breaks apart haplotypes over generations, introgressed haplotypes should be longer than haplotypes due to ILS and should exhibit higher levels of linkage disequilibrium (see figure 1 from Racimo et al., 2015). If admixture occurred recently compared to the divergence between populations, these features can be exploited to detect introgressed tracts. A test of significance can be associated by performing coalescent simulations of specific demographic scenarios (setting values of divergence times, recombination rates, population structure or selection adapted to the case in hand) to obtain the expectations for haplotype length statistics in the absence of gene flow. Haplotype length analyses led to the identification of candidate introgressed tracts and estimation of the age of the last introgression event in humans (Racimo et al., 2015) and dogs (Miao et al., 2016). A recently developed statistic, S^∗^, uses linkage disequilibrium information to detect introgressed haplotypes when no information about the donor is available. S^∗^ is designed to identify divergent haplotypes whose variants are in strong linkage disequilibrium and are not found in a non-admixed reference population. S^∗^ increases as the number of linked SNPs and the distance between them increases (Vernot et al., 2016). This statistic helped to reveal the introgressed origin of the EPAS1 gene in Tibetans, before the identification of the Denisovan donor (Huerta-Sánchez et al., 2014).

### Detection of Selection

To prove adaptive introgression, the action of selection has to be demonstrated on the introgressed variant. A number of reviews address methods and tools for detecting selection with molecular data (e.g., Bank et al., 2014; Pavlidis and Alachiotis, 2017). In practice, most of the available approaches are more sensitive to signatures of strong positive selection (i.e., “hard” selective sweeps, Smith and Haigh, 1974). For regions under strong positive selection, expectations are lower diversity, higher linkage disequilibrium and specific distortions of the allele frequency spectrum compared to the genome-wide patterns.

In within-population analyses, local patterns of lower genome diversity and shifts of the allele frequency spectrum toward an excess of low frequency alleles are often informative for detecting positive selection. For instance, polymorphism summary statistics, such as *π* (nucleotide expected heterozygosity) and Tajima’s *D*, have helped to discover and characterize the introgressed loci involved in serpentine adaptation of *A. arenosa* (Arnold et al., 2016) and in the pesticide resistance of mosquitoes (Norris et al., 2015) and mice (Song et al., 2011). Advanced methods for genome scans of positive selection are the Composite Likelihood Ratio test approaches (reviewed in Pavlidis and Alachiotis, 2017). These tests compare the probability of the observed local site frequency spectrum under a model of selection with the probability of observing the data under the standard neutral model. The neutral expectations can be inferred by genome-wide observed patterns or by specific simulated demographic scenarios (e.g., Staubach et al., 2012; Liu et al., 2015; Quach et al., 2016).

Haplotypic information is also extremely useful for identifying almost fixed or very recently fixed selective sweeps. The frequency of the introgressed haplotype in the recipient population can serve for identifying selection. This interpretation is based on the assumption that introgressed regions under selection should be at higher frequencies in the population relatively to the rest of the genome (e.g., Vernot et al., 2016). The extent of linkage disequilibrium generated on the sides of a beneficial mutation (or the haplotype size) is another signature captured by a number of tests for selection (Crisci et al., 2012). The BADH2 gene, responsible for the much-appreciated characteristic fragrance of some Asian rice varieties, provides a nice example of adaptive introgression detected by haplotype analysis. This gene only shows strong signatures of selection in fragrant accessions, as revealed by a dramatic reduction in diversity (*π*) and a large block of linkage disequilibrium in regions flanking the functional mutation. The selected fragrant allele is likely to have originated after domestication in the genetic background of the *japonica* varietal group and to have been transferred to the *indica* variety by introgression (Kovach et al., 2009).

Extreme differentiation between populations in specific genomic regions can also be interpreted as a signature of selection subtending local adaptation. For introgressed alleles adaptive in the recipient population, higher differentiation can be expected between the recipient and another non-admixed population (e.g., Ai et al., 2015). In addition, recipient–donor differentiation will be lower for introgressed regions compared to the rest of the genome (Figure 1B). Thus, comparisons of pairwise differentiation values between different populations (i.e., donor, recipient and “reference” non-admixed population) may help to disentangle instances of adaptive introgression (e.g., Arnold et al., 2016; Enciso-Romero et al., 2017; Racimo et al., 2017). A number of differentiation/divergence statistics with different properties are available (e.g., Cruickshank and Hahn, 2014). Among them, estimators of *F_ST_* (Wright, 1931) are the most commonly used for detecting selection (e.g., Gagnaire et al., 2009; Arnold et al., 2016; Gittelman et al., 2016). To take advantage of the different sensibilities of each statistic, a useful strategy can be to combine them, as done by Bellucci et al. (2014). These authors used a combined index to include both within and between population statistics to identify selection signals. Note, however, that the value of all these statistics is affected by the demographic history, which can lead to high rates of false positives. Using neutral coalescent simulations to build the distribution of expected value of the statistics in absence of selection is a solid strategy to identify significant outliers (e.g., Bellucci et al., 2014).

It should be noted, however, that inferring separately introgression and selection might not be the best approach to detect adaptive introgression, as the genetic patterns expected in case of selection alone are not always those expected under adaptive introgression. Notably, the loss of diversity typically associated to strong selection may not be found. In this sense, it has been shown by simulations that admixture can increase diversity blowing the diversity loss due to selection (Racimo et al., 2017). Recent investigations into the joint dynamics of introgression and positive selection have opened promising avenues for the analysis of genetic data in quest of adaptive introgression instances (Racimo et al., 2017). These authors proposed new statistics informative to identify candidates to adaptive introgression based on the number and frequency of alleles shared by the donor and the recipient populations (but absent or nearly absent in non-introgressed reference populations). Such “unique shared alleles” should be numerous and at high frequency in genomic regions interested by adaptive introgression (Figure 1C). The proposed statistics resuming these patterns, Q95 and U, have proven successful to retrieve several known regions of archaic adaptive introgression from Neanderthals and Denisovans in modern human genome (Racimo et al., 2017). However, the threshold value of these statistics indicative of adaptive introgression is not straightforwardly transferable among study systems. *Ad hoc* demographic simulations are necessary to assess their expected value in absence of adaptive introgression.

Most of the evidence of adaptive introgression detected so far likely targets instances of strong directional selection. If introgressed adaptive alleles are under types of selection other than hard sweeps, then genetic patterns generated will be more difficult to distinguish and would go undetected with the approaches described above. For instance, balancing selection would favor heterozygosity, thus would maintain introgressed alleles at intermediate frequency within the recipient population. Such a pattern can be interpreted as the result of migration-drift equilibrium, unless a direct link has been established between the locus and a phenotypic trait. Examples of adaptive introgression driven by balancing selection are the incompatibility locus in *Arabidopsis* (Castric et al., 2008), skin color change in wolves (Anderson et al., 2009; von Holdt et al., 2016) and the HLA locus in humans (Abi-Rached et al., 2011).

Selection occurring by soft-sweeps, i.e., the fixation of a beneficial allele starting from multiple copies of it in the population (Hermisson and Pennings, 2005), is also typically difficult to detect. Soft sweeps may occur in two circumstances, either the beneficial allele arises independently multiple times in the population or it is already segregating at certain frequency when it becomes beneficial. In both cases, the beneficial variant rises in frequency associated to different genetic backgrounds. The consequence is that diversity around the selected site and the site frequency spectrum does not change dramatically as in hard selective sweeps (Hermisson and Pennings, 2017), making it hard to detect. Soft sweeps can occur in introgressed regions; in fact, the same beneficial allele can enter into the recipient population associated with different genetic backgrounds when the migration rate is high. Progresses are been made toward the identification of this kind of selection (Flagel et al., 2018).

**FIGURE 3:**
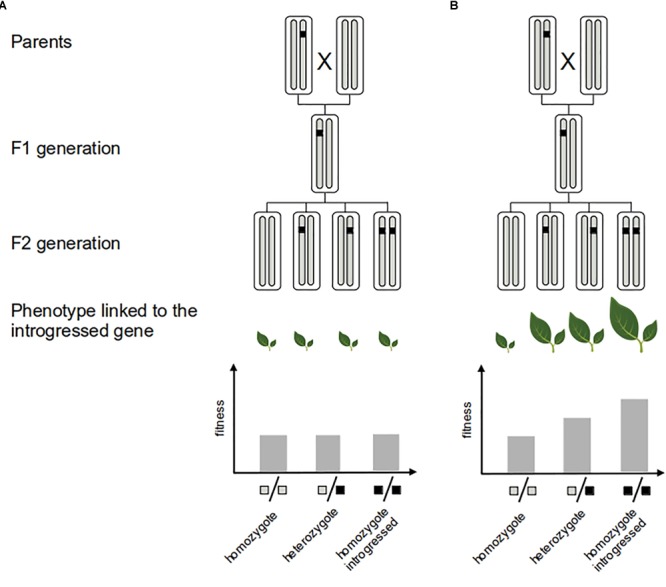
Measure of the fitness of adaptive introgression. Direct evidence of the adaptive value of the introgressed fragment (black segment) consists in showing that it confers greater fitness to the recipient genome. This can be achieved by experimental crosses: neutral introgression **(A)** vs. adaptive introgression **(B)**. Several generations of crosses are needed to generate multiples genotypes with an homogeneous genetic background that allows to quantify the fitness effect of the introgressed fragment.

It is important to point out that inferences of selection based on molecular data only give indirect evidence of the adaptive value of introgression, particularly when they target genomic regions with an unknown contribution to fitness-related traits (e.g., Gagnaire et al., 2009). However, detecting selection in genic regions linked to specific functions or phenotypes (shown by phenotype–genotype association analysis for instance) greatly helps the interpretation in terms of adaptation (e.g., Hufford et al., 2013; Racimo et al., 2015; Rochus et al., 2018). Ultimately, one direct validation of the adaptive role of introgression is to demonstrate the fitness advantage of the introgressed allele or trait for the recipient population (e.g., Martin et al., 2006; Whitney et al., 2006, 2010, 2015) (Figure 3). However, field studies involving phenotypic exploration can be time-consuming and difficult to implement for most species.

## Harnessing Adaptive Introgression in and for Crops Evolution

Anderson (1949) was one of the first highlighting that “cultivated plants and weeds are very largely the products of introgression” and that “from Mendel’s original peas to Blakeslee’s Daturas, we have worked chiefly with introgressed germplasms.”

### Why Humans Matter?

At first stage of domestication, human migration and ancient trade routes have created multiple opportunities of contact between newly locally adapted cultivated or wild forms and thus potentially bringing together previously isolated species/forms (Allaby et al., 2008; Meyer and Purugganan, 2013). Increased resulting variability may have been selected by first farmers, which could even have favored the apparition of weeds (Anderson, 1949). At a later stage in the domestication process, adaptive introgression of agronomic traits between different breeds or cultivars may have occurred relatively easily under similar human selective pressures (e.g., preference for a particular phenotype). In contrast, the transfer of beneficial traits from wild relatives may seems more difficult, even though the genetic divergence between domesticates and wild relatives is usually low and the reproductive barriers narrow (Dempewolf et al., 2012). Crop-wild admixed plants may tend to be phenotypically intermediate and thus to have unwanted wild-type traits (e.g., asynchrony of phenology changes, multiple branching, seed shattering, noxious or unpalatable compounds) that could be counter-selected by farmers. Jarvis and Hodgkin (1999) were among the first to point to the necessity of understanding local farmers’ taxonomy and practices to understand how farmer knowingly or not allow wild alleles to enter the crop gene pool.

In traditional agro-pastoralism systems where all wild species are not discarded, the mixing of cultivated, fallow and pasture areas provide good conditions for introgression to occur. In such systems, harvested seed stock is selected and conserved by farmers for the next year. Barnaud et al. (2009) investigated the criteria used by farmers to characterize the morphotypes of domesticated and weedy sorghum. They found that strong counter-selection was exerted against weedy types, but the progeny of weedy types, i.e., second generation hybrids and more, could be misidentified as cultivated forms and be conserved thus favoring wild-to-crop introgression. For pearl millet, it has been suggested that incomplete weeding and singling allow hybridization and introgression to occur freely and extensively (Couturon et al., 1997; Robert et al., 2003; Mariac et al., 2006), favoring the maintenance of wild genetic material in the cultivated gene pool. In some circumstances, weedy types are voluntarily conserved, because they play an important role for food security. Usually, weedy types are early maturing plants more resistant to harsh conditions, thus they may constitute a key resource in unfavorable years or between main harvests. In Sudan, farmers recognize a crop-wild hybrid of sorghum, which is allowed to grow and is selectively harvested in bad years (Ejeta and Grenier, 2005). Hybrids can be harvested during periods of scarcity also in the case of pearl millet (Couturon et al., 2003; Mariac et al., 2006) and common bean (de la Cruz et al., 2005; Zizumbo-Villarreal et al., 2005). Some studies have also documented farmers practicing conscious directional selection toward changes of cultivated phenotypes by using the diversity available in the wild relatives. For instance, in Benin, farmers deliberately grow wild and hybrid yams (*Dioscorea* spp.) in their fields to increase diversity (Scarcelli et al., 2006). This is also the case of the Ari people of Ethiopia, which aim at increasing the diversity of ensete (false banana, *Ensete ventricosum*) landraces by crossing them with wild populations (Shigeta, 1990). Overall, this demonstrates how farmer practices can maintain and, in some cases, actively favor wild-to-crop introgression.

Farmers’ practices can also facilitate the introgression of improvement traits from modern cultivars into landraces. This is the case, for instance, of Indian farmer’s seed management. By favoring introgression of modern cultivars into their landraces farmers were able to improve yield without losing landrace drought tolerance (Vom Brocke et al., 2003). Another interesting example is provided by the Italian maize landraces. Taking advantage of 50 years temporal sampling, Bitocchi et al. (2015) were able to show how gene flow with introduced cultivars reshaped the genetic diversity of landraces. While some loci were suggestive of adaptive introgression, others where strongly counter-selected, suggesting that different parts of the genome had differential permeability to gene flow, as mentioned below.

Overall, it is important to stress out that adaptive introgression has higher opportunities to occur in (i) traditional farming systems where landraces are used and farmers select seed stocks over generations and (ii) low input systems where wild resistance traits could favor crop fitness (crop higher fitness). In intensive systems with no nutrient/water limitation and use of pesticides, it would be more difficult to identify and thus select for wild advantageous alleles.

### Conditions for Wild Adaptive Introgression Into the Crop Gene Pool

It may be wondered to what extent introgression from wild relatives can affect the whole crop genome, in other words, which is the probability of wild introgression into the crop gene pool. Recent studies have suggested that introgression can be favored at genome-wide level when it reduces the genetic load of the recipient species (Sankararaman et al., 2014; Wang et al., 2017; Kim et al., 2018). Genetic load refers to the genome-wide accumulation of weakly deleterious alleles that reduces its fitness (Crow, 1989). Crops experience a reduction in fitness compared to the wild progenitor that is known as ‘cost of domestication’ (Lu et al., 2006). Given the repeated selection rounds associated with the domestication process, crop species experience a reduction in the effective population size and in effective recombination, which in turn reduces the efficacy of purifying selection in removing deleterious alleles and increases the effect of hitchhiking selection (i.e., deleterious variants increase in frequency because they are linked to selected beneficial alleles). Inbreeding, which is commonly practiced to fix traits of interest, also slightly contributes to fixing deleterious alleles. A greater genetic load than in the wild counterpart was observed in several domesticated species such as rice (Lu et al., 2006), maize (Wang et al., 2017), sunflower (Renaut and Rieseberg, 2015), dogs (Marsden et al., 2016), and horses (Schubert et al., 2014). Since wild species are expected to have a lower genetic load than cultivated species, spontaneous introgression from wild species could be favored even in the absence of strong directional selection, because it alleviates the domestication cost. Recent findings in maize support this expectation, as negative correlations were observed between wild introgression and genetic load (Wang et al., 2017).

The reduction of genetic load by introgression would concern the whole genome. However, different parts of the genome are expected to be differentially permeable to gene flow. In particular, regions involved in major domestication characters are expected to be under strong human selection, thereby acting as barriers to gene flow (so-called “islands of domestication,” Frantz et al., 2015). The efficacy of human selection against introgression would depend on the genetic distance between the introgressed fragment and the domestication genes, which is determined by the extent of local linkage disequilibrium. According to this expectation, Hufford et al. (2013) identified cold spots and hotspots of wild introgression in the maize genome, and cold spots were significantly enriched in domestication genes (Hufford et al., 2013). The implication is that the probability to find adaptive introgression along the crop genome largely depends on the number and distribution of domestication loci. Loci responsible for domestication traits have been identified in a number of crops (Doebley et al., 2006; Gross and Olsen, 2010; Meyer et al., 2012), but knowledge is far from complete. Up to now, research on the genetic architecture of domestication traits indicates that domestication loci are limited to a few genomic regions in most studied species (Burger et al., 2008; Glémin and Bataillon, 2009) and therefore may not be a major obstacle to introgression in the remaining of the genome.

Other factors may influence the probability of adaptive introgression in crop species. For allopolyploid crops, reproductive isolation from the two parental species might be too strong to allow introgression. Limited adaptive introgression may be also expected for highly autogamous crops, for which the frequency of gene flow from external sources is reduced compared to allogamous species (but when occurring it is expected to fix faster, as proven by rice domestication history). This implies that there is a higher chance for adaptive introgression in diploid and allogamous crops for which reproductive isolation with wild relatives is not complete.

### Challenges of Addressing Adaptive Introgression in Crops

Despite the advances in DNA sequencing technologies and analytical developments (see the section “Characterizing Adaptive Introgression With Genetic Data”), targeting spontaneous adaptive introgression for crop improvement still presents a number of challenges. On one hand, crop wild relative (CWR) genetic resources are largely unexplored, often under-represented in *ex situ* collections and threatened in the wild (Dempewolf et al., 2017). Then, the identification of the parental populations is not straightforward. Besides the lack of genetic and phenotypic knowledge underlined above, accessions currently held in gene banks lack important information, including collection sites. A related major issue is to establish to what extent wild accession are really “wild.” In many cases, molecular studies revealed significant crop-to-wild gene flow (Rendón-Anaya et al., 2017) that increase the wild diversity (Assoumane et al., 2018) and confuse the identification of the wild part of the genome.

Second, as explained above (see the section “Characterizing Adaptive Introgression With Genetic Data”), the low genetic divergence usually observed in crop-wild systems could make difficult to differentiate between introgression and incomplete lineage sorting. Furthermore, crop demographic history is often complex and can confound inferences of selection. However, the field is moving fast toward new analytical approaches designed to target specific features in genomes associated to the adaptive introgression process (e.g., Racimo et al., 2017), which may allow soon improving our ability in the detection of adaptive introgression in spite of confounding factors. In addition, crop species could benefit of an alternative strategy to improve our power to detect adaptive introgression. This consists in analyzing the cultivated gene pool at different times in the past, taking advantage of long-term collections stored in gene banks. By tracking changes of allele frequencies across time, our power to detect selection should increase.

In the end, the validation of adaptive introgression detected via molecular data would need the association of classical experiments to measure the strength of selection in the field (Figure 3) and to assess the biological function of the introgressed alleles (Whitney et al., 2006; Suarez-Gonzalez et al., 2018b). Because of their adaptation to human controlled environments, this step seems easier to accomplish in most crops than in wild species.

### Opportunities for Crop Research and Breeding

Often, spontaneous gene flow and introgression have been largely seen as sources of undesirable consequences in the agronomic context (e.g., weedy invasiveness, transgene escape). We believe instead that more attention should be devoted to the potential of introgression as source of useful adaptations for the domesticated species in crop research. A documented outcome of historical adaptive introgression from wild to crop is the ability to colonize new environments, e.g., high altitude habitats in maize, (Hufford et al., 2013); northern latitudes in potatoes (Rendón-Anaya et al., 2017). It is arguable that spontaneous gene flow from locally adapted sources is helping crop populations to cope with current environmental changes. Therefore, screening for the wild introgression already existing in the cultivated gene pool may be an effective strategy to uncover wild adaptive variants relevant for crop adaptation to environmental changes.

Traits involved in climate and soil adaptation, or resistance to pests and diseases, display much greater diversity in wild than in domesticated populations (Hajjar and Hodgkin, 2007; Guarino and Lobell, 2011; Dempewolf et al., 2017). However, extensively exploiting wild alleles for the improvement of the cultivated gene pool still presents many difficulties. One constant major challenge for the plant breeder is to isolate beneficial transgressive segregates with minimum linkage drag, to avoid the introduction of undesirable traits or reduced agronomical performances (Dempewolf et al., 2017; Hussain et al., 2017). To do so, different pre-breeding approaches can be used, such as sequential backcrossing design in which small regions of the wild relative are introduced (e.g., Cavanagh et al., 2008; McMullen et al., 2009). This approach usually requires to create recombinant lines with complex crossing schemes. In such populations, inbred lines are preferentially used as parents, which implies several selfing steps for both cultivated and wild genotypes. While the interest of this kind of hybrid breeding populations is unquestionable, the drawback is a strongly reduction of the wild diversity introduced and thus investigated. Efforts will likely be done to increase the wild diversity included in this type of approaches. In their study, von Wettberg et al. (2018) stated: “we envision creating lineages bearing key cultivated traits, but that otherwise possess high levels of wild variation. We argue that a collection of such lineages will provide an ideal system in which to test the agronomic utility of the wild backgrounds. Such an approach could be especially powerful in the case of traits conferred by multiple loci of small effect size, for example, removing deleterious mutations that are predicted to accumulate.”

Artificial wild-crop hybrid populations are particularly interesting for polyploide and/or autogamous crops, whose probabilities of receiving spontaneous gene flow are very limited, such as ground peanuts (Fonceka et al., 2012). But for crops with significant outcrossing rates such as maize or pearl millet, such artificial populations are very much similar to naturally introgressed landraces. Screening spontaneous adaptive introgression to identify adaptive wild variants has the advantage that high number of generations of recombination under farmer recurrent selection have already reduced the linkage drag. An additional advantage is that these landraces will also carry large sets of introgressed alleles at multiple loci, potentially allowing adaptation even for polygenic traits (Mallet, 2007; Abbott et al., 2013). In this respect, we could consider introgressed crop populations as already established pre-breeding populations, which can be exploited to tap the wild resources for the improvement of modern cultivars to future climatic conditions. We even have now the ability to assess if such introgression are directly associated with crop adaptive responses to new or more extreme stresses using climatic or ecophysiological models and large-scale phenotyping studies. Investigating adaptive introgression in landraces would be, therefore, an useful complementary approach to the development of recombinant inbred line hybrid populations from wild-crop populations for the improvement of adapted varieties.

Finally, addressing spontaneous introgression in crops, will contribute to the understanding of broad evolutionary questions regarding the interplay of selection and gene flow and the dynamics of genome permeability to introgression. From a conservation and management point of view, this knowledge will contribute to assessing the risk of transgene escapes in the case of crop-to-wild gene flow. Understanding introgression, and adaptive introgression more specifically, could help also in implementing targeted crossing for the genetic rescue of threatened landraces, i.e., to increase their adaptive diversity without losing their specific gene pool.

## Conclusion

Examples of adaptive introgression are accumulating in any kind of organism. Wild-crop gene flow seems to have played an important role in the evolution of modern-day crop diversity, although its implication in crop adaptation has been shown in only few species. In the near future, the popularization of high-throughput genotyping and phenotyping screening, together with the development of appropriate analytical tools, will provide the opportunity to investigate the role and mechanism of spontaneous adaptive introgression in crops. The identification of local adaptive introgression could be an effective way to target the relevant adaptive diversity to be deployed for the development of more sustainable and climate-resilient varieties. To this end, we emphasize the need of conserving the genetic resources of wild populations and traditional landraces, and of routinely including wild relatives in crop research programs.

## Author Contributions

CB, AB, and CB-S wrote the first draft. All authors made a substantial, direct and intellectual contribution to this work.

## Conflict of Interest Statement

The authors declare that the research was conducted in the absence of any commercial or financial relationships that could be construed as a potential conflict of interest.
